# Antivirals blocking entry of enteroviruses and therapeutic potential

**DOI:** 10.1186/s12929-021-00708-8

**Published:** 2021-01-15

**Authors:** Mohd Ishtiaq Anasir, Faisal Zarif, Chit Laa Poh

**Affiliations:** Centre for Virus and Vaccine Research, Sunway University, 5, Jalan Universiti, 47500 Bandar Sunway, Selangor Malaysia

**Keywords:** Antiviral compound, Antiviral peptide, Enterovirus, *Picornaviridae*

## Abstract

Viruses from the genus Enterovirus (EV) of the *Picornaviridae* family are known to cause diseases such as hand foot and mouth disease (HFMD), respiratory diseases, encephalitis and myocarditis. The capsid of EV is an attractive target for the development of direct-acting small molecules that can interfere with viral entry. Some of the capsid binders have been evaluated in clinical trials but the majority have failed due to insufficient efficacy or unacceptable off-target effects. Furthermore, most of the capsid binders exhibited a low barrier to resistance. Alternatively, host-targeting inhibitors such as peptides derived from the capsid of EV that can recognize cellular receptors have been identified. However, the majority of these peptides displayed low anti-EV potency (µM range) as compared to the potency of small molecule compounds (nM range). Nonetheless, the development of anti-EV peptides is warranted as they may complement the small-molecules in a drug combination strategy to treat EVs. Lastly, structure-based approach to design antiviral peptides should be utilized to unearth potent anti-EV peptides.

## Introduction

The genus Enterovirus (EV) belonging to the *Picornaviridae* family comprises 13 species, of which seven are human viruses [1]. Four of the species are: (1) EV-A such as coxsackievirus (CV)-A6, CV-A10, CV-A16 and EV-A71, (2) EV-B such as the CV-B viruses, echoviruses (ECHO) and CV-A9, (3) EV-C such as polioviruses (PV) and CV-A21, (4) EV-D such as EV-D68 and EV-D70 [1]. The other three species are rhinoviruses RV-A, RV-B and RV-C which comprised over 100 different numbered RVs [1]. EV RNA contains a single open reading frame (ORF) flanked by two untranslated regions (UTRs), 5′ UTR and 3′ UTR [2]. The ORF encodes a single polyprotein that is cleaved into P1, P2 and P3 proteins [3]. The P1 protein is proteolytically cleaved to produce capsid proteins VP1–4 [3]. P2 and P3 are cleaved to produce non-structural (NS) proteins 2A, 2B, 2C and 3A, 3B, 3C, 3D, respectively [3]. The role of the capsid proteins is to enclose the genetic material and to recognize cellular receptors during viral entry [3]. The NS proteins are crucial for replication, translation and subversion of host cell machinery [3]. The capsid proteins are suitable targets for antiviral development due to their role in cellular entry and uncoating of the genetic material [3].

The diverse viruses in the genus EV are known to cause a range of diseases such as hand, foot and mouth disease (HFMD), encephalitis, aseptic meningitis, myocarditis and various respiratory diseases [1]. Although most EV infections are mild, the symptoms can be severe in the very young and immunodeficient individuals [4]. In recent years, viruses such as EV-A71 and CV-A16 have emerged as serious public health threats, as they have caused major outbreaks of HFMD in China and South East Asia [5, 6]. Additionally, EV-D68 has caused a large outbreak of severe lower respiratory infections in North America in 2014 [7]. Therefore, broad-spectrum antiviral drugs that could inhibit multiple EVs across the genus will be instrumental to overcome the public health burden caused by these EVs. In this review, we will summarize the efforts in developing direct-acting antivirals targeting the capsid of EVs and host factor-targeting inhibitors. The low barrier to resistance of the capsid binders will be discussed and the possible strategies to overcome this challenge will be suggested. Lastly, we look at the viability of peptide-based strategy to develop anti-EV therapies.

### The architecture of enterovirus capsid

**Fig. 1:**
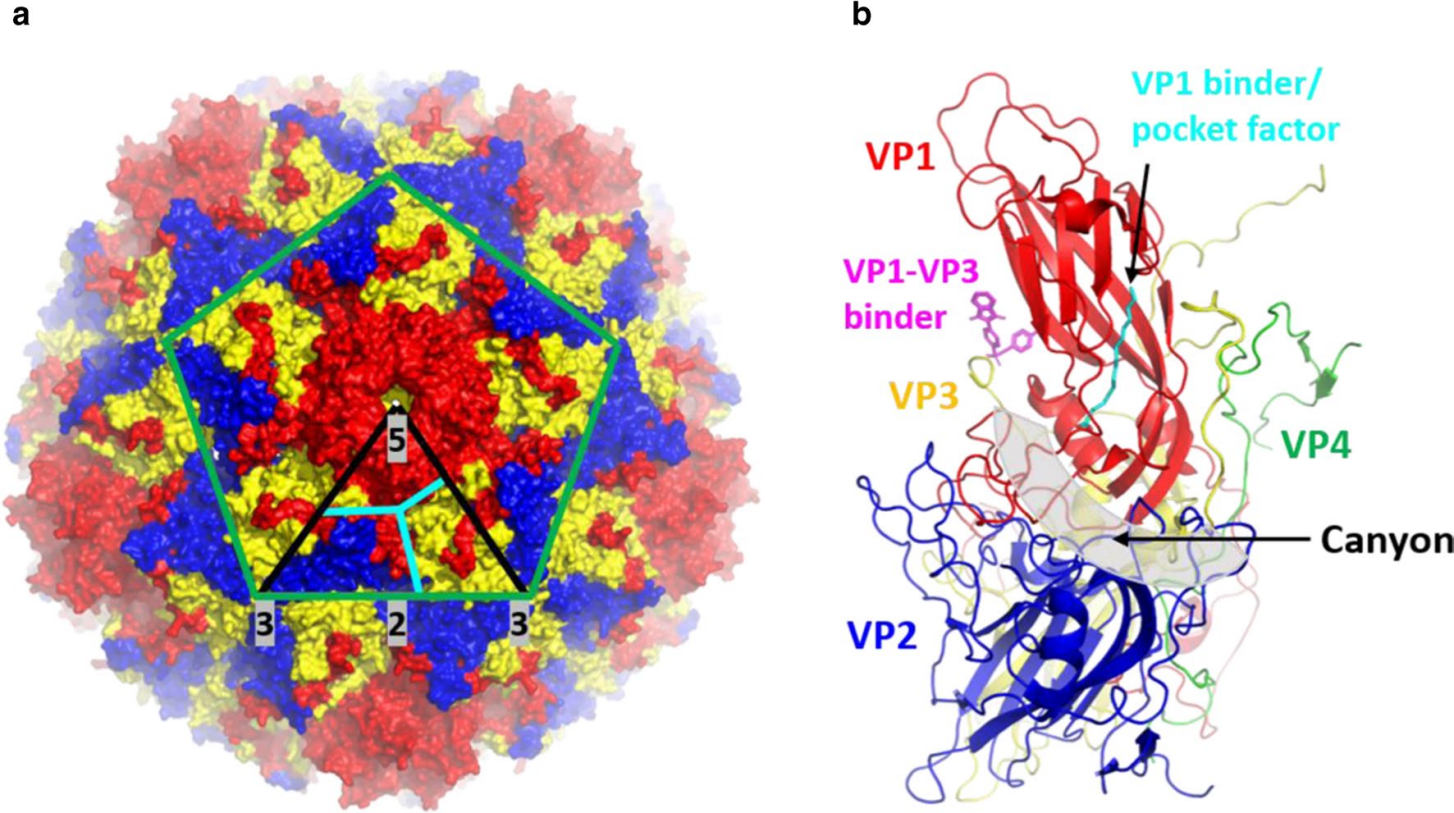
Enterovirus capsid organization and features. **a** Overall view of the enterovirus capsid comprising the VP1 (red), VP2 (blue) and VP3 (yellow). PDB ID: 4RQP [8]. The green lines indicate the boundaries of one pentamer. The black lines indicate the icosahedral symmetric subunit. The five, three, twofold symmetry axes are labeled and highlighted in grey. The cyan lines separate VP1 (red), VP2 (blue) and VP3 (yellow). Black arrows indicate the canyon region and the fivefold axis region formed by five VP1. **b** Canonical enterovirus protomer formed by VP1 (red), VP2 (blue), VP3 (yellow) and VP4 (green). PDB ID: 6GZV [9]. The canyon is highlighted in grey transparent and is indicated by a black arrow. Antiviral compounds that bind to the two binding pockets which are VP1 hydrophobic pocket and VP1–VP3 interprotomer pocket are shown in cyan and magenta, respectively

All EVs have a naked icosahedral capsid with five, three, and twofold rotational symmetry formed by 60 identical protomers (Fig. 1a) [8]. Each protomer is composed of VP1, VP2 and VP3 that formed the capsid surface while VP4 is located in the inner surface of the capsid [1]. VP1 to VP3 have a common fold formed by eight-stranded β barrels and two α helices [1]. The main surface features of the external capsid include: (1) star-shaped surface protrusions formed by five copies of VP1, (2) a “canyon” formed by the junction of a “north rim” formed by VP1 and “south rim” formed by VP2 and VP3 encircling the fivefold axes, (3) a protrusion or “puff” formed by VP2 loop, (4) a “knob” formed by a VP3 loop, (5) a large twofold depression and (6) VP1 hydrophobic pockets beneath the canyon bound by lipid molecules known as “pocket factors” (Fig. 1a, b) [1, 8, 9]. The differences in the loops connecting the α helices and β barrels result in the unique surface capsid traits between different EVs [1, 3].

### Enterovirus cellular attachment and uncoating

EV infections start with viral attachment to cellular receptors [10]. The majority of the cellular receptors belong to the (Ig) superfamily or the integrin receptors [10]. EVs recognize cellular receptors by accommodating the apical Ig domain into the canyon (Fig. 1b) [9]. In contrast, non-Ig fold receptors are recognized by EVs via regions outside of the canyon, such as the vertex of the fivefold axis of viral capsid [11]. The viral canyon-host receptor binding usually triggers the formation of an expanded particle known as the altered (A) particle primed for genome uncoating [12–14]. On the other hand, the interaction of cellular receptors with regions outside of the canyon seldom induce significant conformational changes. Instead, this interaction has been shown to signal for the localization of the attached virus to the main receptors that can bind to the canyon region [15]. For example, CVB3 binds to the co-receptor decay-accelerating factor (DAF) to facilitate the localization of the attached virus to its main receptor, coxsackie and adeno receptor (CAR) [15]. However, some EVs such as EV-A71, CV-A10 and CV-A16 have a shallow canyon indicating that they employ a distinct mechanism for receptor binding and uncoating [16]. Indeed, one of the uncoating receptors for EV-A71 and CV-A16 is the human scavenger receptor class B member 2 (SCARB2) which is a non-Ig fold receptor that binds mainly to the VP1 GH and VP2 EF loops instead of the canyon region [17–19].

Following receptor binding, EVs will empty their pocket factors from the VP1 hydrophobic pockets [10]. This expulsion triggers a cascade of conformational changes to form the A particles characterized by radial expansion and pore formation [1, 20]. The pores allow the release of myristylated VP4 which is inserted into cellular membranes and the exposure of the hydrophobic N-terminus of VP1 which facilitates the shackling of the virion to the cell membranes [1, 21]. These events promote the transport of the viral genome into the cytosol. Currently, our understanding of the mechanism of RNA release is still limited. The 3D structure of an uncoating intermediate of a clinical C4 strain of EV-A71 indicated that a significant capsid rearrangement at the icosahedral two and fivefold axes allowed the formation of large channels for the release of viral RNA [21]. More recently, cryo-electron microscopy study of human ECHO18 and 30 revealed that the release of RNA from the viral particles requires the loss of one, two or three adjacent capsid pentamers [20]. Therefore, stabilization of the pentamers by small molecules or peptides could be a viable strategy to inhibit genome release.

## Enteroviral drugs evaluated in clinical trials

One of the highly explored strategies to hinder EV infections is to target their capsids. There are three regions on the EV capsid that have been identified to be viable targets for drug development. The first is the VP1 hydrophobic pocket occupied by the pocket factor [22]. Many direct-acting antivirals targeting this pocket have been identified (Table 1) [23]. These compounds dislodge the pocket factor and bind to the hydrophobic pocket to stabilize the capsid in a rigid and compressed form [23]. This prevents the formation of expanded A particles that is required for genome uncoating [24]. Additionally, there are evidence that demonstrated the binding of compounds to this pocket hindered EV attachment to host cells [25]. Generally, the hydrophobic pocket binders inhibited EV infectivity with half-maximal inhibitory concentration (IC_50_) or half-maximal effective concentration (EC_50_) in the nM to pM ranges [26–32]. Five compounds that have been evaluated in phase I and II clinical trials were disoxaril, pleconaril, pirodavir, vapendavir and pocapavir [33–38]. Despite their promising in vitro potencies, the majority of these inhibitors demonstrated insufficient efficacy and unwanted side effects in clinical trials. The unwanted side effects include asymptomatic crystalluria seen in patients receiving disoxaril and the induction of cytochrome P-450 3A (CYP3A4) enzymes by pleconaril that led to menstrual irregularities in pleconaril-treated women taking oral contraceptives [33, 34].

**Table 1:** Antivirals targeting the enteroviral proteins evaluated in clinical trials

Compound	Enterovirus inhibited	In vitro potency	Clinical trial results	Refs
Capsid binders				
Disoxaril	EV-B, C, D70, RV-A, B	nM-μM	The clinical studies were halted due to the appearance of crystalluria in healthy individuals	[33, 44]
Pleconaril	EV-B, C, D68, RV-A, B	nM-μM	FDA application for RV colds rejected due to safety concerns	[29]
Pirodavir	EV-A, B, C, D, RV-A, B	pM-nM	No clinical benefit in treating RV colds	[35, 45]
Vapendavir	EV-A71, C, D68, RV-A, B	nM	Failed to reduced asthma exacerbations in a phase II clinical trial	[46]
Pocapavir	EV-B, C	nM	Accelerated the clearance of monovalent oral PV1 vaccine in healthy adults	[31, 32, 38]
3C protease inhibitors				
Rupintrivir	All	nM	Failed to show significant beneficial effects in clinical trials for RV common colds	[39, 47]
AG7404	EV-A, B, C, D, RV-A, B	nM-μM	Failed to show significant beneficial effects in clinical trials for RV common colds	[40, 48]
3A and/or 3AB inhibitor				
Enviroxime	EV-A, B, C, D, RV-A, B	nM-μM	Clinical development was discontinued due to insufficient therapeutic effects and gastrointestinal side effects	[41, 43, 44]

In addition to the capsid binders, the 3C protease inhibitors such as rupintrivir and its analog AG7404 have also been evaluated in clinical trials (Table 1). The 3C proteases are essential for cleaving the polyprotein precursor into structural proteins and non-structural proteins responsible for viral replication. However, these inhibitors failed to show significant beneficial effects in clinical trials involving RV [39, 40]. Enviroxime is another compound that has been evaluated in clinical trials [41]. It inhibited EV infections by targeting the viral proteins 3A and/or 3AB to prevent the formation of the replication complex [42]. Despite showing potent EV replication inhibition in vitro, its clinical development was halted due to gastrointestinal side effects and the lack of therapeutic effect [41, 43, 44].

### Anti-enterovirus targeting the VP1 hydrophobic pocket

Despite the failures, significant efforts are being put into identifying novel compounds that target the VP1 hydrophobic pocket (Table 2). For instance, Kim et al. [49] identified a novel series of benzothiophene derivatives and analogues with potent antiviral activities against RV-A and RV-B strains. In particular, compound 6g inhibited RV-A21 (EC_50_: 0.078 µM), RV-A71 (EC_50_: 0.015 µM) and RV-B14 (EC_50_: 0.083 µM) [49]. It also inhibited PV3 (EC_50_: 0.063 µM), indicating the potential of these compounds to inhibit other EVs as well [49]. Molecular docking study demonstrated the subtle difference between the binding modes of 6g and pleconaril whereby 6g formed a distinct hydrophobic interaction between its 3-methyl group and Leu25 in VP3 [49]. In addition, PR66 which is an imidazolidinone derivative was found to inhibit the uncoating process of EV-A71 by interacting with the VP1 hydrophobic pocket [50]. PR66 was demonstrated to provide complete protection in mice against neurological symptoms induced by EV-A71 [50].

**Table 2:** Small molecule compounds targeting viral capsid

Compound	Enterovirus inhibited	Potency (EC_50_ or IC_50_)	Refs
VP1 hydrophobic pocket			
Compound 6g	RV-A21, RV-A71, RV-B14, PV3	nM	[49]
PR66	EV-A71	nM	
ALD	EV-A71	nM	[51]
NLD	EV-A71	pM	[51]
ICA135	CV-A10, EV-A71, CV-A16, CV-B3, PV1 and EV-D68	nM-µM	[16]
Compound 10g	CV-B3, RV-A, RV-B	nM-µM	[74]
Fivefold vertex			
Suramin	EV-A and EV-B (CV-A9, ECHO20, ECO25)	µM	[60]
NF449	EV-A71	µM	[61]
E151	EV-A71, CV-A6, CV-A16	µM	[63]
Dendrimer 12	EV-A71, ECHO11, EV-D68	pM-µM	[64, 65]
HS mimetics	EV-A71	µM	[68]
HS fragments	EV-A71	µM	[69]
Rosmarinic acid	EV-A71	µM	[70]
VP1–VP3 interprotomer			
Compound 12	Specific for CV-B1, CV-B3, CV-B4, CV-B5, CV-B6	µM	[72]
Compound 1	Specific for CV-B1, CV-B2, CV-B3, CV-B4, CV-B5, CV-B6	µM	[72]
Compound 17	CV-B1, CV-B3, CV-B6, CV-B4, CV-B5 and CV-A9	nM-µM	[9]

*HS* Heparan sulfate

Structure-based rational design of VP1 hydrophobic pocket binders has also been pursued. The structural analysis of four pyridyl imidazolidinones derivatives (GPP2, GPP3, GPP4 and GPP12) in complexes with EV-A71 facilitated the design of two highly potent anti-EV-A71 compounds ALD and NLD with notable IC_50_ values of 8.5 nM and 25 pM, respectively [51]. Both compounds also inhibited a wide range of other EVs including CV-A9, CV-A16, CV-A21, CV-B3, PV1-3, RV-2 and RV-14 with IC_50_ values ranging from pM to µM [52].

### Antivirals targeting the fivefold axis of the capsid

The second region that can be targeted by antiviral compounds is the fivefold axis of the capsid. Many of the EV-A members such as CV-A6, CV-A16 and EV-A71 and EV-B members like CV-A9 and ECHO5 possess the positively charged fivefold axis that is responsible for viral attachment to host cell receptors including PSGL1 and heparan sulfate [53–57]. However, McLeish et al. [55] demonstrated that ECHO6 did not bind to heparan sulfate despite having a positive charge cluster at the fivefold axis. This suggests precise structure and conformation of the positive cluster is critical for the interaction between the fivefold axis and host receptors [55]. As SCARB2 was demonstrated to be the main attachment and uncoating receptor for EV-A viruses [58], it can be speculated that its inhibition could prevent EV infection. However, there is no report of any antiviral agent capable of inhibiting the binding of EVs to SCARB2.

Various compound series have been identified to target the fivefold axis (Table 2). One of the compounds, suramin, is a multi-functional molecule that has been evaluated for potential applications in viral diseases and cancer, despite its manifold adverse effects which have been reported including nephrotoxicity and dermatitis [59]. Ren et al. [60] reported that suramin inhibited several EV-A viruses including CV-A2, 3, 10, 12, and 16, and some EV-B viruses such as CV-A9, ECHO20 and ECHO25. Suramin and its derivatives such as NF449 were proposed to interact with the fivefold axis of the capsid to prevent EV association with PSGL1 and heparan sulfate [60, 61]. In vivo studies revealed that suramin significantly reduced mortality in mice challenged with a lethal dose of EV-A71 and decreased the peak viral load in adult rhesus monkeys [62].

Screening of sulfonated azo dyes against EVs has shown that the majority of the dyes exhibited in vitro inhibitory effects on the infectivity of EV-A71. In particular, brilliant black BN (E151) inhibited three EVs which are EV-A71, CV-A6 and CV-A16 [63]. It had the highest efficacy in blocking virus entry and it protected AG129 mice against EV-A71 lethal challenge. However, the in vitro potency of E151 is low with IC_50_ values ranging from 2.39 to 28.12 µM for various EV-A71 strains. E151 was identified to interact with the fivefold axis of the capsid and inhibited PSGL1 and cyclophilin A (CyP-A)-mediated EV-A71 entry into host cells [63].

Furthermore, the attachment of EV-A71 to host cells via PSGL1 and heparan sulfate was reported to be inhibited by a series of tryptophan dendrimers that target the fivefold axis of EV capsid [64, 65]. These dendrimers contain different central scaffolds and multiple tryptophan groups that are linked to the dendrimer branches through an amino group. A consensus compound named dendrimer 12 that was synthesized according to the structure–activity relationship analysis of the series was found to inhibit a large panel of EV-A71 clinical isolates with high potency in the nM to pM range [64].

The anti-EV activities of heparan sulfate mimetics have also been evaluated since a number of in vivo studies in mice and monkeys have demonstrated heparan sulfate could specifically interact with the key residue VP1-145G in EV-A71 to inhibit the virus [66, 67]. The mimetics including heparin, heparan sulfate and pentosan polysulfate were shown to exhibit antiviral actions against EV-A71 with low potency in the µM range [68, 69]. In addition, shorter heparan sulfate-based fragments exhibited inhibitory actions against EV-A71 infection [69]. These compounds bind to the capsid of EV-A71 to act as decoy receptors to block viral attachment [69]. A comparison of the in vitro potency between the small molecules and the larger mimetics revealed that the former exhibited a higher potency than the latter [69]. For instance, compound 22 has an IC_50_ value of 8.5 µg/mL, in comparison to IC_50_ values of 102.1 µg/mL and 142.8 µg/mL for heparan sulfate and heparin, respectively [69]. Importantly, the shorter heparan sulfate disaccharide mimetics lacked anti-coagulant activities and will not cause unwanted side effects [69].

Rosmarinic acid (RA) which is a compound from herbal medicine *Salvia miltiorrhiza* (Danshen) was found to target this region as well [70]. Similar to most of the compounds targeting this region, RA inhibited various EV-A71 genotypes with IC_50_ values in the μM range (Table 2) [70, 71]. In vivo evaluation revealed that RA reduced the mortality of mice infected with mouse-adapted EV-A71 strain [70, 71].

### VP1–VP3 interprotomer binding pocket

More recently, a novel druggable pocket within the conserved VP1–VP3 interprotomer interface of the viral capsid has been reported (Table 2) [9, 72]. The novel drug target was initially identified in screening the antiviral activity of 4-dimethylamino benzoic acid (compound 12) and its analogues [72]. Compound 12 displayed weak potency with an EC_50_ value of 9 μM while its most potent analogue compound 1 displayed antiviral activity with an EC_50_ of 2.6 μM against CV-B3. These compounds were identified to be highly specific against CV-B viruses as they did not inhibit other EVs such as ECHO11, EV-A71, RV-2 and RV-14. Mutational and molecular modeling studies revealed that compound 12 and its analogues bind to a small cavity surrounded by amino acids Arg219 and Tyr75 from two different units of VP1 of CV-B3, which is distinct to the VP1 hydrophobic pocket targeted by pleconaril. Furthermore, the evaluation of the combinatorial antiviral activity of compound 12 and pleconaril revealed that the mechanism of action of compound 12 is distinct from that of pleconaril, indicating that both drugs targeted different binding pockets.

Subsequently, a benzenesulfonamide derivative, compound 17, was identified as an inhibitor of CV-B3 with an EC_50_ value of 0.7 μM [9]. It also inhibited the replication of CV-B1, CV-B6, CV-B4, CV-B5 and CV-A9. However, it lacked activity against viruses in the EV-A group (CV-A16 and EV-A71), EV-C group (CV-A21 and PV1), EV-D group (EV-D68) and RV-B group (RVB14). Structural study of compound 17 interaction with the viral capsid revealed that the compound binds to a pocket formed by two VP1 units (amino acids 73, 75–78, 155–157,159–160, 219, and 234) and one VP3 unit (amino acids 233–236) at the interface of an interprotomer. This novel drug target is located 16 Å away from the VP1 hydrophobic pocket. Sequence analysis revealed that the pocket is reasonably conserved across the EV-B group, with 7 of 16 amino acids being identical across the eight CV-B viruses including CV-B1, CV-B2, CV-B3, CV-B4, CV-B5, CV-B6, CV-A9 and ECHO11. Furthermore, the binding site is also conserved across a panel of EVs, in particular the amino acids Arg219 and Arg234 of CV-B3 [73]. The underlying mechanism of EV inhibition by compounds targeting this pocket is yet to be fully elucidated. It was proposed that the binding of compounds in this pocket stabilized the viral particle, which ultimately impeded structural rearrangements that allowed the transition to the A-particle [73].

Abdelnabi et al. [9] initiated hit optimization to develop a broad-spectrum antiviral that could inhibit the replication of multiple EV groups. The skeleton of compound 17 was used as the core structure to design more active analogues. The medicinal chemistry efforts guided by the data from antiviral assays yielded a series of broad-spectrum analogues with activity against EV-B (CV-Bs), EV-C (PV1 and CV-A21), EV-D (EVD68), RV-A (RVA09, RVA59, and RVA63), and RV-B (RVB14). However, the analogues still lacked activity against EV-A viruses such as CV-A16 and EV-A71. Among the broad-spectrum analogues include compound 48 with activities against EV-B and EV-C viruses and compound 77 with activities against RV-A and RV-B groups. Some of the compounds were also active against echoviruses E1 and E7.

### The emergence of resistant variants towards antiviral drugs

Antiviral resistance is the major drawback for all the direct-acting antivirals targeting the EV capsid. In some cases, a single amino acid substitution within the binding pocket of EV was sufficient to reduce or completely abolish the antiviral activity of the capsid binders [75]. Many in vitro studies have demonstrated that the propagation of EV in the presence of capsid binders would lead to the emergence of resistant variants [30, 37, 76–78]. For example, serial passaging of EV-A71 and CV-A16 in the presence of ALD or NLD led to mutations in the VP1 of EV-A71 (Ile113 and Val123) and CV-A16 (Leu113) [78]. The amino acids were identified to be substituted with bulkier amino acids such as Ile113Met and Val123Ile in resistant EV-A71 and Leu113Phe in resistant CV-A16. The bulky amino acids hindered the entry of these inhibitors into the VP1 hydrophobic pockets. Resistant variants have also been identified in individuals receiving treatment during clinical trials. For instance, the clinical trials to evaluate pleconaril efficacy to treat cold symptoms in RV-infected individuals have indicated that RVs with reduced susceptibility to pleconaril were identified from 10.7% of the pleconaril-treated patients [79]. Additionally, fully pleconaril-resistant RVs were also recovered from 2.7% of these patients [79].

Although the capsid binders exhibited a low barrier to resistance, the majority of the resistant variants displayed lower fitness and virulence than the wild-type. For instance pleconaril-resistant RV isolated from patients appeared to be non-pathogenic and attenuated in cell cultures [79]. In addition, escape mutants such as NLD-resistant EV-A71 and CV-A16 variants were found to readily revert to the wild-type genotype when passaged in the absence of NLD [78].

### Strategies to overcome drug resistance

Multiple strategies have been pursued to overcome the drug resistance problem. Combining antiviral agents with synergistic antiviral effects is a proven approach to increase antiviral potency, exemplified by the success in the combinatorial treatment regimens of human immunodeficiency virus (HIV) and hepatitis C virus (HCV) [80, 81]. Additionally, the use of antiviral agents with different mechanism and resistance profiles creates a higher barrier to genetic mutations, thereby hindering the emergence of resistance. Wang et al. [82] have demonstrated that the combination of two anti-EV drugs, rupintrivir and itraconazole, was shown to reduce the risk of generating drug-resistant EV-A71 mutants. Studies to investigate the in vivo combinatorial effects of anti-EV drugs such as disoxaril/guanidine/oxoglaucine and pleconaril/MDL-860/oxoglaucine in newborn mice infected with coxsackieviruses revealed that the combinations of these drugs prevented the development of drug resistance against the capsid binders [83–85].

Another strategy that has been explored to overcome antiviral resistance is by modifying the physical properties of existing antiviral agents. The study of structure–activity relationships has facilitated the selection of compound scaffolds that can facilitate the design of new inhibitors with limited antiviral resistance and unwanted side effects [74, 86]. For instance, the pleconaril scaffold has been used as the basis for the development of novel compounds such as the orally available compound 10g which could inhibit pleconaril-resistant EVs with IC_50_ values between 0.02 and 5.25 µM [74]. Additionally, compound 10g is a weaker inducer of CYP3A4 enzymes that pleconaril, lowering the risk of off-target effects [74].

## Peptide-based anti-enteroviral development

Peptide-based strategy is another viable approach to develop anti-EV drugs especially with the success of the FDA-approved antiviral peptide drug enfuvirtide. Enfuvirtide is a peptide derived from a region within the human immunodeficiency virus (HIV-1) glycoprotein 41 (gp41) [87]. It inhibits HIV-1 infection by blocking the membrane fusion between HIV-1 and cellular membranes. The peptide-based strategy possesses several advantages over small-molecule compounds as they are easy to synthesize, exhibit a higher barrier to viral resistance and have a lower toxicity. In addition, peptides are better at targeting pockets that are too large to be occupied by small-molecule compounds. A study indicated that compounds that partially occupy the VP1 hydrophobic pocket exhibited weaker potency than larger compounds that have better occupancy [51]. However, larger chemical compounds are associated with difficulty to synthesize, high cost of production and poor bioavailability.

Multiple peptides have been identified to inhibit EV-A71 and other enteroviruses (Table 3). In a study by Tan et al. [88], four peptides SP40, SP45, SP55 and SP81 derived from the VP1 of EV-A71 were found to inhibit EV-A71 with the SP40 peptide exhibiting the highest antiviral activity with an IC_50_ of 6 µM. Synergistic antiviral activity assays revealed that SP40, SP45 and SP55 peptides might exert their activities against EV-A71 by inhibiting the viral attachment in the early phase of the infection. In contrast, SP81 exerted its activity at a later stage of EV-A71 infection viz*.* at post-viral entry [89]. Furthermore, SP40 peptide was shown to be active against other viruses in EV-A and EV-C groups such as CV-A16 and PV1, respectively [88].

**Table 3:** Antiviral peptides against enteroviruses

Peptide	Sequence	Potency (EC_50_ or IC_50_)	EV inhibited	Refs
SP40	Ac-QMRRKVELFTYMRFD-NH_2_	6–9.3 µM	EV-A71, CV-A16, PV1	[88]
SP45	Ac-AEFTFVACTPTGEVV-NH_2_	N/A	EV-A71	[88]
SP55	Ac-PESRESLAW-NH_2_	N/A	EV-A71	[88]
SP81	Ac-SKSKYPLVVRIYMRMKHVRAW-NH_2_	N/A	EV-A71	[88]
LVLQTM	LVLQTM	nM–µM	EV-A71, Echo-6, RV-2	[90, 91]
Tripeptide VAD	VAD	µM	RV-2	[92]
Tetrapeptide AAPV	AAPV	µM	PV-1, CV-A21, RV-2	[93]

*N/A* not available

Another peptide (LVLQTM) that acted as a pseudosubstrate to the 2A protease was found to inhibit multiple EV infections [90]. This peptide binds to the active site of 2A protease to reduce its activity with an IC_50_ value of 0.3 μM [91]. Various other peptides have been shown to target 2A proteases from various EVs such as the tripeptide VAD and tetrapeptide AAPV with IC_50_ values of 5.6 μM and 20–65 μM, respectively [92, 93]. In general, the majority of the anti-EV peptides displayed lower potencies (> 0.3 μM) in comparison to the small-molecules with in vitro efficacies in the nM to pM range. Nevertheless, the development of anti-EV peptides is warranted as these peptides may be utilized in a combinatorial drug approach together with the small-molecules. Optimization of the peptides such as cholesterol tagging may improve the potency of these peptides, in particular the peptides targeting host proteins at the cellular membrane such as SP40 peptide.

### Design of antiviral peptides targeting the canyon region on the surface of enteroviruses

Despite the wealth in structural information of the EV capsids, no direct-acting antiviral or host-targeting inhibitor has been designed using the structure-based drug design approach. There are several regions on the EV capsids that can be targeted by peptides. For instance, the atomic structure of EVs in complex with their receptors may guide the design of antiviral peptides derived from the complex interface on the receptors [18]. These peptides may act as direct-acting antivirals by interacting with the regions on the capsid and act as decoys to competitively inhibit EV attachment to host cells.

**Fig. 2:**
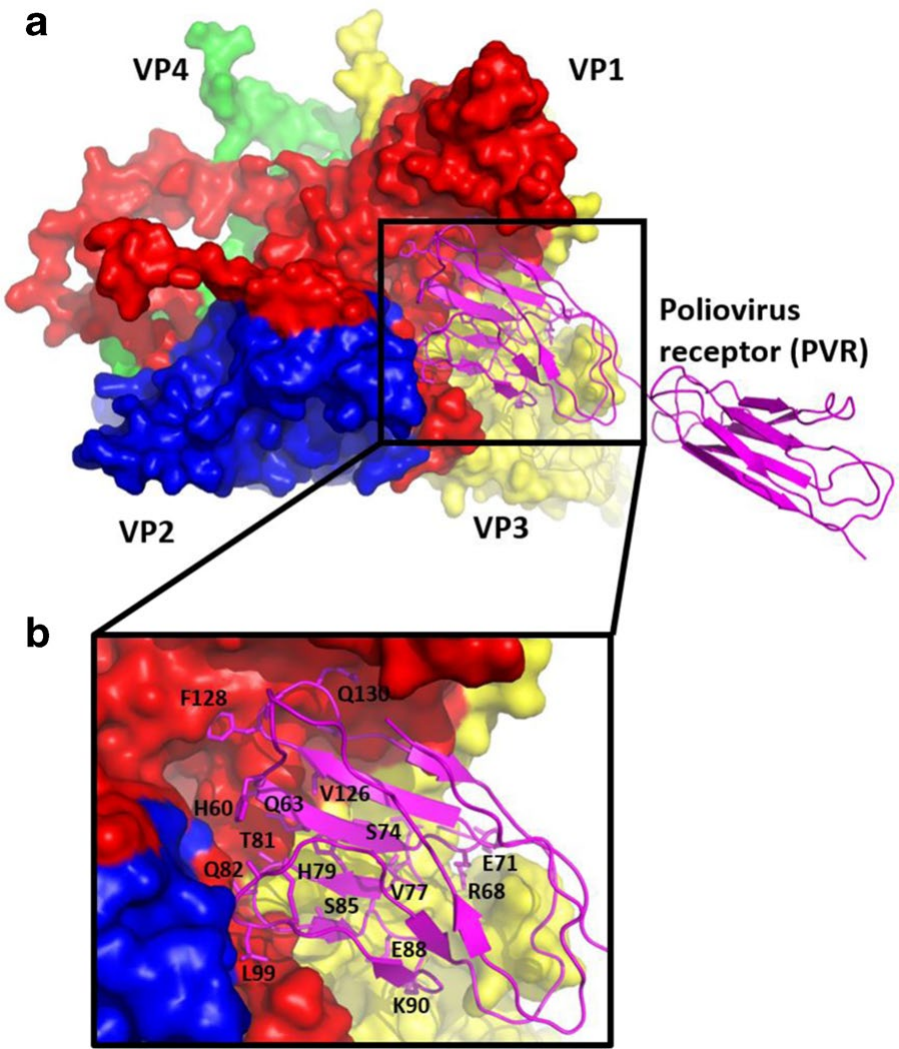
The structure of poliovirus in complex with PVR (PDB ID: 3EPD) [94]. **a** Overall view of the canonical picornavirus protomer with the capsid proteins VP1, VP2, VP3 and VP4 are shown in red, yellow, blue and green, respectively. The apical domain of PVR that binds to the canyon of the protomer is shown magenta. **b** The amino acids that make contacts with the canyon are shown in sticks and labeled

Taking poliovirus in complex with PVR as an example, the PVR binds to the quasi-threefold axis region contacting with three capsid proteins VP1, VP2 and VP3 [12]. Structural analysis revealed that the canyon region of the PV capsid formed ionic and hydrophobic interactions with two regions spanning amino acids 60 to 99 and 126 to 130 within the apical domain of PVR (Fig. 2). Therefore, peptides derived from these two regions could potentially bind to the canyon and hinder the attachment of poliovirus to the PVR. There are several advantages associated with utilizing the peptide-based strategy to target the canyon region on the EV capsid surface. Firstly, targeting this region using peptides is advantageous as the peptides can engage the virus surface extracellularly. This removes the need to consider the permeability of the peptides. Secondly, peptides that act on the virus may display lower cytotoxicity than antivirals targeting host proteins that are prone to cause unwanted off-target effects. Lastly, capsid proteins are highly conserved among viral family, therefore they are promising to be developed as broad-spectrum antivirals against multiple EV infections.

### Design of antiviral peptides targeting the cellular receptors

**Fig. 3:**
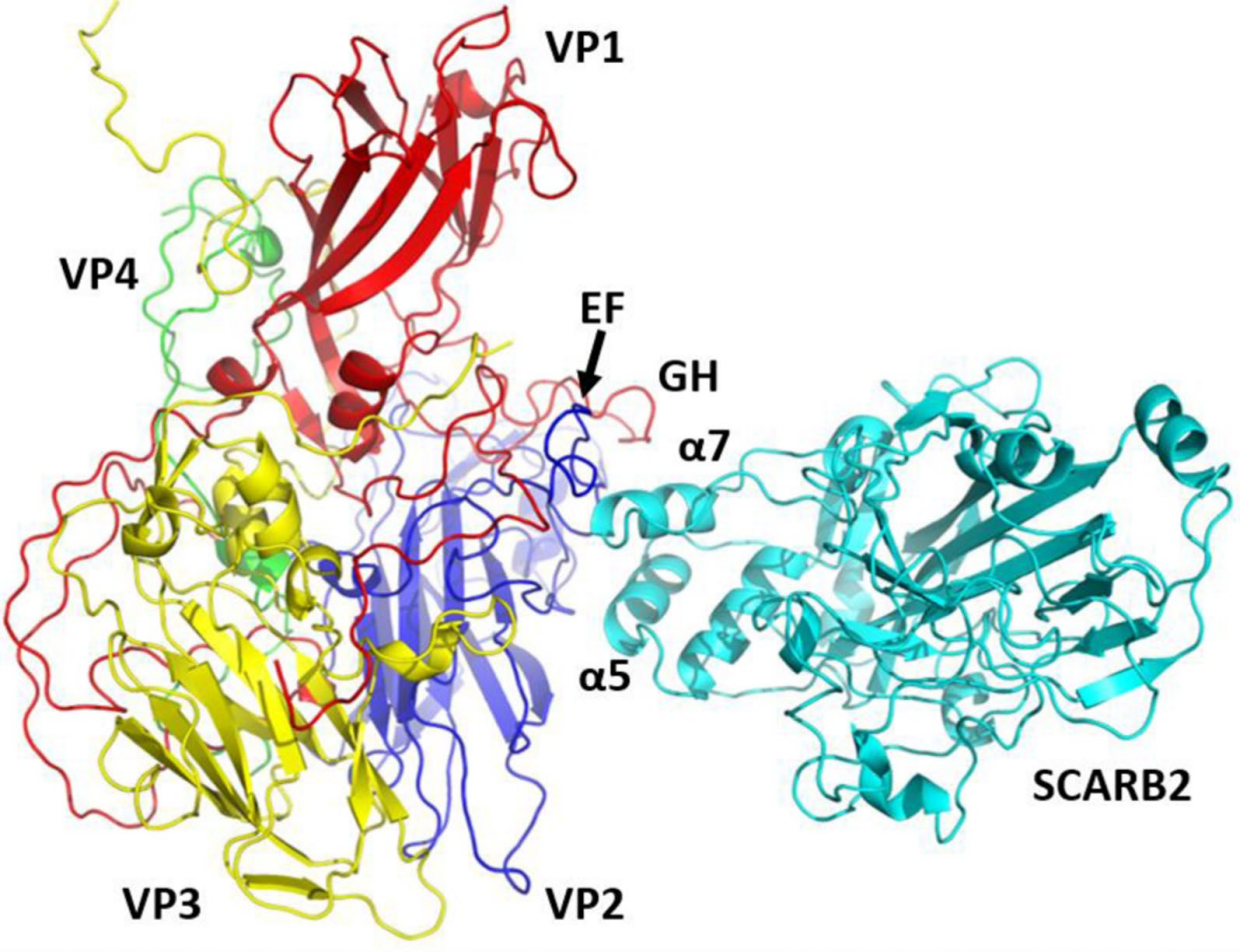
Three-dimensional structure of EV-A71 in complex with SCARB2 (PDB: 6I2K) [18]. The EV-A71 capsid proteins VP1, VP2, VP3 and VP4 are shown in red, yellow, blue and green, respectively. The receptor SCARB2 is shown in cyan. VP2 EF loop and VP1 GH loop of EV-A71 interact with α5 and α7 helices of SCARB2 to form the interface of the complex

Apart from designing direct-acting antiviral peptides, the structural information of EV in complex with host receptors can be utilized to design peptides that can target host proteins, in particular the cellular receptors [81, 95]. Taking the structure of EV-A71 in complex with SCARB2 as an example, peptides derived from the capsid region can be evaluated for their ability to prevent viral attachment to host cells (Fig. 3) [18]. Structural analysis revealed that the interface of the EV-A71:SCARB2 complex is formed by the α5 (aa 152–163) and α7 (aa 183–193) helices of SCARB2 and VP1 GH and VP2 EF loops of EV-A71 [18]. Peptides mimicking the GH loop of VP1 and EF loop of VP2 could potentially bind to SCARB2, blocking the interactions between EV-A71 and SCARB2. There are several advantages of targeting the host cellular receptors [81]. Host-targeting antivirals generally possess a higher barrier to resistance than its virus-targeting counterparts [96]. In addition, antiviral peptides targeting the host receptor could provide a broad inhibition of multiple viruses from different genotypes and serotypes and possibly other viruses in the *Picornaviridae* family that utilize the same receptor for cellular attachment [97].

### Limitations of peptide-based antiviral strategy

Despite the advantages of antiviral peptides, several limitations remain to be addressed. First, antiviral peptides generally exhibited a weaker potency than small-molecule compounds [81]. This issue could be resolved by modification strategies such as cholesterol tagging that have been proven to increase the potency of antiviral peptides. Cholesterol tagging was shown to enhance the local concentration of peptides at the membrane and improved membrane permeability [81, 98, 99]. Second, poor bioavailability and short half-life are common limitations for peptide-based strategy since peptides are susceptible to cleavage by peptidases and proteases [99]. The use of D-amino acids could decrease recognition and binding of peptides to proteolytic enzymes [100]. Terminal capping by post-translational modifications such as N-terminal acetylation and C-terminal amidation could enhance the ability of peptides to resist degradation by exopeptidases [101]. Furthermore, encapsulation into nanoparticles could increase the stability and bioavailability of antiviral peptides [102]. Lastly, there is an issue with the high production cost related to peptide synthesis and purification [99, 103]. Factors such as expensive reagents and low purity of final products coupled with challenging processes to introduce disulfide bridges in certain peptides significantly increased the production cost [99, 103]. Effective peptide production methods like the use of recombinant expression systems in hosts such as bacteria and yeast could significantly reduce the cost of peptide production [104, 105].

## Conclusions

The eradication of EVs is challenging because these viruses are not easily inactivated and may survive well in water and sewage for long periods [106]. Therefore, the development of vaccines and antivirals should be pursued to mitigate EV epidemics. The ultimate goal of anti-EV research is to develop safe and effective antiviral agents without generating drug-resistant EVs. Many inhibitors targeting the surface capsid of EV have been identified with five of them being evaluated for their safety and efficacy in clinical trials. However, the majority of the inhibitors were found to cause unwanted side effects and failed to meet their clinical endpoints despite exhibiting potent in vitro activity in the nM to pM range. Furthermore, the inhibitors are prone to generate resistant variants, albeit the resistant variants exhibited reduced fitness in comparison to their wild-type counterparts. Nonetheless, researchers should explore the strategies such as drug combination therapy and drug optimization based on the structure–activity relationships to improve antiviral potency and increase the resistance barrier of the inhibitors. Lastly, the peptide-based antiviral strategy should be explored either as an alternative or to complement the anti-EV small-molecules.

## Data Availability

Not applicable.
